# National trend of gastric cancer mortality in China (2003–2015): a population-based study

**DOI:** 10.1186/s40880-019-0372-x

**Published:** 2019-05-02

**Authors:** Kun Gao, Jun Wu

**Affiliations:** 1 Key Laboratory of Biomedical Information Engineering of Ministry of Education, School of Life Science and Technology, Xi’an Jiaotong University, Xi’an, 710049 Shannxi P. R. China; 2 Department of Pharmaceutical and Administrative Sciences, Presbyterian College School of Pharmacy, 307 North Broad Street, Clinton, SC 29325 USA

**Keywords:** Gastric cancer, Mortality, Age-standardized rate, Rural population, Urban population, Health service

## Abstract

**Background:**

Gastric cancer mortality decreased substantially over the last decades in most countries worldwide. This study aimed to assess the most recent national trend of gastric cancer mortality and examine the disparity of gastric cancer mortality between rural and urban areas in China.

**Methods:**

The crude mortality data of gastric cancer by sex, age group, and area were obtained from China Health Statistical Yearbooks (2003–2015) covering 10% of Chinese population. The age-standardized rates of mortality (ASRM) of gastric cancer in rural and urban areas were estimated using the 2010 Chinese Census population stratified by age, sex, and area. The trend of mortality of gastric cancer was assessed by using Joinpoint analysis.

**Results:**

During the 13-year period, the ASRM was reduced from 31.5/100,000 in 2003 to 20.9/100,000 in 2015 in rural areas and from 18.9/100,000 in 2003 to 14.5/100,000 in 2015 in urban areas. In the male population, the annual percent changes of mortality were − 2.2% in urban areas (95% confidence interval [CI] − 3.8% to − 0.6%; *P* < 0.001) and − 3.4% in rural areas (95% CI − 5.1% to − 1.8%; *P* < 0.001). In the female population, the annual percent changes of mortality were − 2.7% in urban areas (95% CI − 4.2% to − 1.2%; *P* < 0.001) and − 4.6% in rural areas (95% CI − 5.5% to − 3.7%; *P *< 0.001).

**Conclusions:**

The declining trend of mortality of gastric cancer was presented from 2003 to 2015 in both rural and urban areas in China. The decrease in gastric cancer mortality is greater in rural areas than in urban areas in China.

## Background

Gastric cancer is a common cancer in China with an age-standardized 5-year survival rate of 27.4% [1, 2]. A previous epidemiological study using regional clinical data have shown that the median survival of gastric cancer patients in China increased from 33 months in 1980 to 49 months in 2003, suggesting a declining trend of gastric cancer mortality [3]. However, gastric cancer deaths in China accounted for about 50% of gastric cancer deaths worldwide [4]. Previous studies described the long-term survival of gastric cancer in China by using data before 2005 [2, 5]. The knowledge of the national trend of gastric cancer mortality during the most recent 10 years is limited. As the world’s largest developing country, China is still characterized by large rural–urban disparities in health care. Due to limited access to health care and poor quality of health service delivered in rural areas, geographic disparity of cancer survival exists between rural and urban areas in China [2]. The disparity in the trend of gastric cancer mortality between rural and urban areas at a national level population has not been well described.

Understanding the most recent trend of gastric cancer mortality stratified by geographic area would help policymakers assess the effectiveness of health policy implemented to prevent and control gastric cancer, identify barriers to access gastric cancer treatment, and ultimately optimize health resource allocation to improve health outcomes for gastric cancer patients. The objectives of this study were to examine the recent trend of gastric cancer mortality using national population data (2003–2015) and to compare the trends between rural and urban areas in China.

## Methods

### Data source

The crude mortality data of gastric cancer by age group were derived from China Health Statistical Yearbooks (2003–2015), as one part of China Economic and Social Development Statistics Database to reflect the development of health services in China and health of residents [6]. The data were collected by the Center of Health Information Statistics under the National Health Commission of China (formerly known as Ministry of Health of China) through national routine death reporting system covering 10% of the Chinese population. The causes of death were classified by International Classification of Diseases, tenth revision (ICD-10) codes in the database. The annual disease-specific mortality in each 5-year age group in the database was reported by sex and area (urban and rural). From 2003 to 2015, the urban samples were selected from metropolitan divisions (ranging from 16 to 17) and principal cities (ranging from 20 to 24) in China. The rural samples were selected from 80 to 90 micropolitan and town areas in China.

### Age-standardized rates of mortality (ASRM)

We estimated annual deaths of gastric cancer by using the 2010 Chinese Census population stratified by age, sex, and area to calculate ASRM of gastric cancer in rural and urban areas for male, female, and whole populations.

### Statistical analysis

Joinpoint regression models developed by Division of Cancer Control and Population Sciences in US National Cancer Institute (Bethesda, MD, USA) [7] were applied to measure the trend of ASRM of gastric cancer. The annual percent changes (APCs) in mortality (urban vs. rural areas) in male, female, and whole populations (2003–2015) were assessed. The number of Joinpoints reflects the significant changes in the trend of mortality for the study population. The statistical significance was set at *P* < 0.05.

## Results

### ASRM by sex and area

**Fig. 1:**
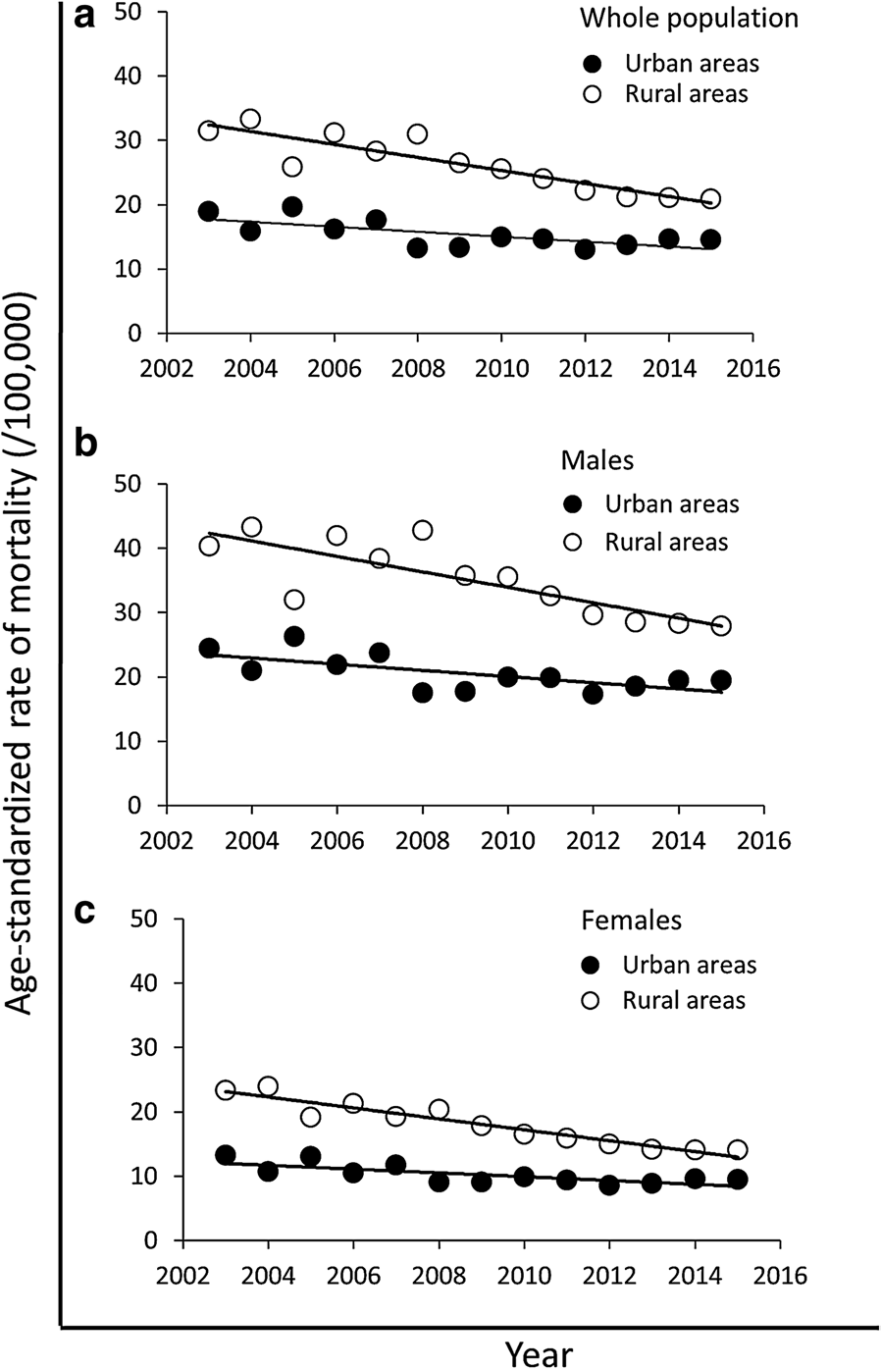
Age-standardized rates of mortality (ASRM) of gastric cancer in rural and urban areas in Chinese populations from 2003 to 2015. ASRMs of gastric cancer were estimated by using the 2010 Chinese Census population for the whole population (**a**), males (**b**), and females (**c**) in rural and urban areas. The scattered data points represent the observed ASRMs. The trend lines were estimated by modeling ASRMs from Joinpoint analysis

Figure 1a shows the trend of ASRM of gastric cancer in the whole population from 2003 to 2015. In rural areas, the ASRM was reduced from 31.5/100,000 in 2003 to 20.9/100,000 in 2015. In urban areas, the ASRM declined from 18.9/100,000 in 2003 to 14.5/100,000 in 2015. Overall, the ASRM of gastric cancer was higher in rural areas than in urban areas during the study period. However, the difference in ASRM between rural and urban areas decreased from 12.6/100,000 in 2003 to 6.4/100,000 in 2015.

Figure 1b shows the trend of ASRM of gastric cancer in the male population. The ASRM of gastric cancer for the male population declined from 24.4/100,000 in 2003 to 19.4/100,000 in 2015 in urban areas and from 40.3/100,000 in 2003 to 27.9/100,000 in 2015 in rural areas. The area disparity was observed during the study period with the gaps of 15.9/100,000 in 2003 and 8.5/100,000 in 2015.

Figure 1c shows the trend of ASRM of gastric cancer in the female population. The ASRM of gastric cancer for the female population was decreased from 13.2/100,000 in 2003 to 9.5/100,000 in 2015 in urban areas and from 23.3/100,000 in 2003 to 14.0/100,000 in 2015 in rural areas. The ASRM of gastric cancer was higher in rural areas than in urban areas with the difference varying from 10.1/100,000 in 2013 to 4.5/100,000 in 2015.

### Trends of gastric cancer mortality

Table 1 presents APC of ASRM of gastric cancer (2003–2015) assessed with Joinpoint regression analysis. The linear trend of mortality was significant with no Joinpoint for male and female populations as well as rural and urban areas in the whole population. In the whole population, the APC of mortality of gastric cancer was − 2.3% in urban areas (*P* < 0.001) and − 3.8% (*P* < 0.001) in rural areas. In the male population, the APC of mortality was − 2.2% in urban areas (*P* < 0.001) and − 3.4% in rural areas (*P* < 0.001). In the female population, the APC of mortality was − 2.7% in urban areas (*P* < 0.001) and − 4.6% in rural areas (*P* < 0.001).

**Table 1:** Annual percent change of age-standardized rates of mortality of gastric cancer in China from 2003 to 2015

Population	Annual percent change (%) (95% CI)	*P*
Whole		
Urban areas	− 2.3 (− 3.9, − 0.8)	< 0.001
Rural areas	− 3.8 (− 5.0, − 2.6)	< 0.001
Male		
Urban areas	− 2.2 (− 3.8, − 0.6)	< 0.001
Rural areas	− 3.4 (− 5.1, − 1.8)	< 0.001
Female		
Urban areas	− 2.7 (− 4.2, − 1.2)	< 0.001
Rural areas	− 4.6 (− 5.5, − 3.7)	< 0.001

*CI* confidence intervals

## Discussion

The present study examined the ASRM of gastric cancer in rural and urban areas by using China national population data (2003–2015). We found declining trends of gastric cancer mortality in both rural and urban areas. For both sexes, the APC of gastric cancer mortality in rural areas was greater than that in urban areas.

A declining incidence of gastric cancer in China (from 22.06/100,000 in 2012 to 19.62/100,000 in 2014) was observed in recent studies [8–10]. In the present study, the annual gastric cancer mortality decreased by 3.8% and 2.3% in rural and urban areas from 2003 to 2015, respectively. Declining trend of gastric cancer mortality could be associated with gradual increase in gastric cancer screening test in China. Early treatment initiation, understanding gastric cancer in depth, and development of technology might contribute to declining trend of gastric cancer mortality [11, 12]. First, although the nationwide screening program has not been fully developed in China, the selective screening programs for high-risk populations have been implemented in the regions with a high incidence of gastric cancer. The detection rate of early gastric cancer varied from 60% to 90% [13, 14]. Screening young adults for *Helicobacter pylori* infection followed by treatment in those who test positive has the potential to prevent 1 in every 4–6 cases of gastric cancer in China and has been shown cost-effective [15]. Since more gastric cancer cases were identified at early stage by screening test, initiation of early treatment could improve clinical outcomes and ultimately reduce mortality significantly. The early gastric cancer detection improved the 5-year survival rate from 63.7% to 89% [16]. Second, declining trend of gastric cancer mortality might be associated with advanced understanding of gastric cancer. The reported findings in previous studies evaluating intermediate outcomes [17, 18] could help clinicians identify high-risk populations to prevent gastric cancer, choose an optimal strategy to treat the disease effectively, and reduce mortality by controlling disease progression. The applications of new technologies to diagnosis and treatment, such as multimodality strategies, improved the outcomes of gastric cancer [19]. For example, adjuvant radiochemotherapy following surgery increased overall survival rate by 32% and relapse-free survival rate by 51% [20]. Addition of perioperative chemotherapy to surgery reduced mortality by 25% and disease progression by 34% [21]. The combination of trastuzumab and chemotherapy became a promising option to treat advanced gastric cancer [22].

Improved awareness of preventing gastric cancer could contribute to the declining trend of gastric cancer incidence. Cancer has been one of the primary causes of death in China. Environment change associated with industrialization and urbanization and rising aging population have an impact on lifestyle change in China. With the prevalent health education delivered by health care providers in communities across the country, more and more people have been motivated to live with a healthy lifestyle to minimize risk factors associated with cancer occurrence [23]. People with high risk factors of gastric cancer, such as *Helicobacter pylori* infection, family history, and unhealthy lifestyle, could be more likely to request screening test and subsequently to start early treatment if gastric cancer is detected at early stage [24].

The gastric cancer mortality gap between rural and urban areas in China existed for both sexes between 2003 and 2015. First, limited access to high-quality health care could explain the overall higher mortality of gastric cancer in rural areas. People in rural areas tend to visit doctors only when symptoms are developed. Thus, the delayed diagnosis and treatment might contribute to higher mortality in rural areas than in urban areas. Improving gastric cancer awareness through health education and expanding screening test to achieve early diagnosis and initiate early treatment could be considered by policymakers to reduce the gap of gastric cancer mortality between rural and urban areas [25]. Second, the present study indicated that the decrease in ASRM in rural areas was greater than that in urban areas. The national gastric cancer screening tests and early treatment intervention were initiated in rural areas in China in the middle of 2000s [26]. This national program helped lower gastric cancer mortality in rural areas significantly. Finally, with the urbanization in China since late 1990s, more and more people have migrated from rural to urban areas and been classified as urban residents. Due to the low socioeconomic status of urban migrants, a large amount of those migrants cannot afford health care in urban areas and kept a high mortality of gastric cancer, which could offset the declining mortality in urban areas. The urbanization might be the potential reason to explain the less decrease in gastric cancer mortality in urban areas than in rural areas from 2003 to 2015. The reform of health care system is needed to provide urban migrants affordable preventive care and treatment.

Some limitations of the present study should be addressed. First, the mortality data were from 1/10 national population. However, it is unknown how rural and urban areas were sampled in the data set. Second, all mortality data were collected from death certificates, and the details of the gastric cancer mortality were not available, such as cancer stage and treatment received. Thus, the causal relationship between early diagnosis through screening test and declining trend of mortality cannot be established. Additionally, the China Health Statistical Yearbooks categorized the disease-specific mortality by using ICD-10 codes. However, the specific ICD-10 codes of gastric cancer were not provided in the data source to differentiate subtypes of gastric cancer. Finally, the China Health Statistical Yearbooks report aggregated gastric cancer mortality by age, sex, and area. The information related to data quality control was not released to help assess the reliability and validity of the database.

## Conclusions

The mortality of gastric cancer declined from 2003 to 2015 in both rural and urban areas in China. The decrease in gastric cancer mortality in rural areas is greater than that in urban areas.

## Abbreviations

ASRM: age-standardized rates of mortality
ICD-10: International Classification of Diseases, tenth revision
APC: annual percent change
CI: confidence intervals
